# Intratracheal Keratinocyte Growth Factor Enhances Surfactant Protein B Expression in Mechanically Ventilated Preterm Pigs

**DOI:** 10.3389/fped.2021.722497

**Published:** 2021-09-28

**Authors:** Ramesh Krishnan, Esmond L. Arrindell, Caminita Frank, Zhang Jie, Randal K. Buddington

**Affiliations:** ^1^Department of Pediatrics, University of Tennessee Health Science Center, Memphis, TN, United States; ^2^Baptist Women's Hospital, Memphis, TN, United States; ^3^Dräger Medical Inc., Telford, PA, United States; ^4^Department of Pathology and Laboratory Medicine, University of Tennessee Health Science Center, Memphis, TN, United States; ^5^Department of Molecular and Cellular Physiology, LSU Health Sciences Center, Shreveport, LA, United States

**Keywords:** preterm, lung injury, surfactant, keratinocyte growth factor, ventilation

## Abstract

Bronchopulmonary dysplasia (BPD) is a devastating disease of prematurity that is associated with mechanical ventilation and hyperoxia. We used preterm pigs delivered at gestational day 102 as a translational model for 26–28-week infants to test the hypothesis administering recombinant human keratinocyte growth factor (rhKGF) at initiation of mechanical ventilation will stimulate type II cell proliferation and surfactant production, mitigate ventilator induced lung injury, and reduce epithelial to mesenchymal transition considered as a precursor to BPD. Newborn preterm pigs were intubated and randomized to receive intratracheal rhKGF (20 μg/kg; *n* = 6) or saline (0.5 ml 0.9% saline; control; *n* = 6) before initiating 24 h of ventilation followed by extubation to nasal oxygen for 12 h before euthanasia and collection of lungs for histopathology and immunohistochemistry to assess expression of surfactant protein B and markers of epithelial to mesenchymal transition. rhKGF pigs required less oxygen during mechanical ventilation, had higher tidal volumes at similar peak pressures indicative of improved lung compliance, and survival was higher after extubation (83% vs. 16%). rhKGF increased surfactant protein B expression (*p* < 0.05) and reduced TGF-1β (*p* < 0.05), that inhibits surfactant production and is a prominent marker for epithelial to mesenchymal transition. Our findings suggest intratracheal administration of rhKGF at initiation of mechanical ventilation enhances surfactant production, reduces ventilator induced lung injury, and attenuates epithelial-mesenchymal transition while improving pulmonary functions. rhKGF is a potential therapeutic strategy to mitigate pulmonary responses of preterm infants that require mechanical ventilation and thereby reduce the incidence and severity of bronchopulmonary dysplasia.

## Background

Despite advances in mechanical ventilation, the mortality, morbidity, and childhood disability associated with bronchopulmonary dysplasia (BPD) remains unchanged due to the increasing numbers of extremely preterm infants surviving birth before 28 weeks of gestation that require mechanical ventilation. Exposing the immature lungs of extremely preterm infants to a combination of the shear stress of intermittent positive-pressure ventilation that causes ventilator-induced lung injury (VILI) (1, 2) and hyperoxia to maintain targeted blood gases (3) decreases production of surfactant, alters expression of pulmonary growth factors, and initiates the pathophysiologic changes of BPD. Decreasing the incidence and severity of BPD requires intervention that will accelerate recovery and restore normal lung growth and development when VILI occurs.

Damage to the pulmonary epithelium triggers proliferation of alveolar type II (ATII) cells that play a key role in alveolar epithelium repair by providing additional ATII cells or by transdifferentiating into ATI cells (4). However, differentiation of ATII cells into fibroblasts through epithelial to mesenchymal transition (EMT) promotes fibrosis and abnormal development of lung tissue that are characteristic of BPD. The pathogenesis of BPD is associated with disturbances of the patterns of expression for the numerous growth factors and associated signaling pathways that are responsible for normal development of the fetal and preterm lungs (5). Notably, mechanical ventilation and hyperoxia disturb secretion of the transforming growth factor (TGF)-beta superfamily of growth factors, leading to EMT, disrupting lung development, and causing lung fibrosis that are hallmarks of BPD (4, 6). Exposure of the preterm lung to increased TGF-β also inhibits proliferation of ATII cells and decreases surfactant expression.

Interest in identifying biomarkers of BPD has revealed disturbances in expression of various other growth factors (7). Keratinocyte growth factor (KGF), also known as fibroblast growth factor (FGF)-7 and a recombinant form is marketed as palifermin, stimulates proliferation, migration, survival, and surfactant production of ATII cells, and increases the clearance of alveolar fluid. *Ex vivo* studies with mature lungs have shown KGF regulates epithelial cell proliferation and surfactant expression (8) and pretreatment reduces the magnitude of damage and diminished pulmonary functions caused by injurious tidal ventilation (9). The potential importance of KGF in mesothelial-epithelial interactions during development is inferred from expression of KGF by mesenchymal cells and restriction of the conjugate receptor to epithelial cells of fetal lungs (10). The reduced expression of KGF associated with mechanical ventilation may involve the VILI induced increase of TGF-β that inhibits KGF expression (11). The decreased KGF can be expected to result in fewer and less functional ATII cells, hinder restoration of the damaged epithelium, and thereby contribute to the pathogenesis of BPD. Despite the advances in understanding KGF from using hyperoxic neonatal rodent models of BPD, the potential benefits of administering exogenous KGF have not been evaluated after extremely preterm birth when mechanical ventilation is required and there is risk of VILI.

Considering the role of KGF in lung development, the reduced expression in response to mechanical ventilation, and the inverse relationship between KGF concentrations in tracheal aspirates from preterm infants and the incidence and severity of BPD (12), we hypothesized administration of exogenous KGF at initiation of mechanical ventilation after preterm delivery would stimulate surfactant production, lower FiO2, and reduce VILI and the EMT that contribute to fibrosis and BPD. This was tested using extremely preterm pigs as a translational model for 26–28-week preterm infants that require invasive mechanical ventilation. Newborn extremely preterm pigs exposed to 24 h of intermittent positive pressure ventilation develop VILI (13) and represent an alternative to using preterm baboons that are mechanically ventilated to induce damage to the epithelium (14). Furthermore, preterm pigs allow for comparisons of littermates without the confounding biological and environmental variables (e.g., different genetics, gestational ages, body weights, intrauterine environments and exposures, and variable postnatal care and disease progression) that confound interpretations of clinical trials.

## Materials and Methods

### Source of Preterm Pigs and Initial Care

The Institutional Animal Care and Use Committees of the University of Tennessee Health Science Center (location of cesarean section) and the University of Memphis (site of ventilation and critical care) approved the protocols for the harvest, care, and sampling of preterm pigs (*Sus scrofa*). Antenatal steroids were not provided, and preterm pigs were delivered via cesarean section on gestational day 102 (89% of 115-day term) from two specific pathogen-free, artificially inseminated sows obtained from a production herd with consistent genetics. After suctioning and clearing the airway, the pigs were placed in a 38–39°C incubator with supplemental oxygen. After spontaneous breathing was established, the pigs were placed in a warmed transport carrier with supplemental oxygen provided by masks that fit over the snout and transported to a neonatal intensive care unit developed for the care of preterm pigs (pNICU). Preterm pigs at this stage of gestation are capable of spontaneous breathing but 80% or more will develop respiratory distress within 6–8 h after delivery and further survival requires invasive mechanical ventilation.

### Instrumentation, Processing, and Intensive Care of Preterm Pigs

The pigs were weighed in the pNICU. An umbilical catheter (UAC; 3.5F Argyle TM, Covidien, MA) was inserted via one of the two umbilical arteries. The UAC was advanced to the descending aorta, and the position was confirmed by radiography (Duoview high Resolution Digital Radiography System, Revo2, Kennesaw, GA). The UAC was used to provide parenteral nutrition (PN), sample arterial blood, and administer Cefazolin (50 mg/kg/dose) as a prophylactic antibiotic. Maternal plasma (5 ml/kg) was also administered via the UAC to provide passive immunity and compensate for the absence of colostrum.

From each of the two litters, 6 pigs of similar body weights that were spontaneously breathing were randomly allocated to 3 controls and 3 treatments. The pigs were initially supported by blow by oxygen and mask and subsequently intubated within approximately 3 h after delivery using red rubber 2.5 French cuffed endotracheal (ET) tubes (Jorgensen Laboratories, Loveland, CO) that minimize leaks during mechanical ventilation (<10%). The position of the ET tubes was confirmed using digital radiography and repositioned, if necessary. The pigs were connected to Dräger VN500 ventilators (Dräger Medical, Incorporated, Dräger, Telford, PA) with initial assist control volume guarantee (AC+VG) settings of a tidal volume of 5 cc/kg, a respiration rate of 40 breaths per min, positive-end expiratory pressure (PEEP) of 5 cm H_2_O, iTime 0.35 seconds, and FiO_2_ of 40%. Surfactant was not administered as it would be difficult to distinguish between endogenous and exogenous sources.

Within 1 h after ventilation was established the pigs were randomized to either the KGF treatment (3 per litter) or sham/control group (3 per L). rhKGF (ProSpec Protein Specialists, Ness Ziona, Israel) produced by E. coli as a single, non-glycosylated polypeptide chain was reconstituted in normal saline. A single dose (20 μg/kg) of 1 ml was divided into two equal aliquots that were administered via the ET tube to each side of the lung followed by hand bagging (PIP: 15 cm H_2_O; PEEP: 5 cm H_2_O) to enhance distribution throughout the lungs. The control pigs received a similar volume of normal saline followed by bagging. Heart rate, oxygen saturation, and perfusion index were monitored continuously (Masimo Radical 7, Masimo, Irvine, CA). Arterial blood gas measurements (iSTAT^®^, Abbott, Abbott Park, IL) were performed after placement of the UAC and every 3 h or after adjustment of peak inspiratory pressure (PIP), ventilation rate, and FiO_2_ to maintain pulse oximetry saturation of 92–98%, pH of 7.25 to 7.4, pCO_2_ 40 to 55 mmHg, and pO_2_ > 60 mmHg during the 24 h of AC+VG The ventilators automatically recorded mean airway pressure, PIPs, tidal volumes, and FiO_2_ every 5 min. The pigs were repositioned each hour from one side to the other to avoid dependent edema. All study pigs were allowed to spontaneously breathe during the study and were not paralyzed. Pigs that became excessively active during the 24 h of mechanical ventilation were sedated to effect using ketamine (Bioniche Teoranta, Galway, Ireland) via the UAC. After 24 h the pigs were extubated and provided supplemental oxygen by nasal cannula for 12 h. At the end of the 36-h study period, all surviving pigs were euthanized (Euthasol; Virbac AH, Inc., Fort Worth, TX, 1 ml/kg; IV).

PN was provided continuously at a rate of 4 ml/kg-h, beginning immediately after placement of the UAC. For the first 4 to 6 h, the pigs received a low potassium (2 mmol) PN solution that provided (per L) 116 g dextrose, 60.5 g amino acids (Travasol), and 31.3 g lipid (Intralipid 30%) with electrolytes, vitamins, and trace elements. Thereafter, a PN solution with normal potassium (5 mmol) was provided. Supplemental fluid was provided via the UAC as needed to maintain tissue perfusion using lactated Ringers and averaged 3–4 ml/kg-h, with the same relative volume administered to all pigs to avoid possible differences caused by variable fluid volumes. The volume of fluid administered was insufficient to cause significant pulmonary edema. Metabolic acidosis as indicated by a base deficit of greater than 10 based on arterial blood gases was corrected using a bolus of normal saline 10 ml/kg with sodium bicarbonate (4.8%; 1 meq/kg) given over 20 min.

### Necropsy

The lungs were collected from pigs that died prior to 36 h and from the remaining pigs that were euthanized after the 24 h of mechanical ventilation followed by the 12 h of supplemental oxygen provided by nasal cannulation. The lungs were removed en bloc and inflated using the ET tube and a NeoPuff™ (Fisher & Paykel Healthcare, Irvine, CA) to a PIP of 20 cm H_2_O. The trachea was clamped, and the right lower lobe was tied off, excised, and submerged in formalin for routine histology and immunohistochemistry (IHC).

### Histologic Analysis

Formalin-fixed tissues were processed in paraffin, embedded in paraffin, and sectioned (4 μm). For routine histology, the sections were stained with hematoxylin and eosin. A pediatric pathologist (JZ) who was blinded to the study protocol semi-quantitatively graded inflammation, hemorrhage, edema, necrosis, and atelectasis of each lung and provided an average for at least five regions. Each parameter was individually scored using a Likert scale from 0 (no injury), 1 (25% injury), 2 (50% injury), 3 (75% injury), and 4 (100% injury) (15).

### Immunohistochemistry

For IHC, the sections were deparaffinized, rehydrated with graded ethanol and treated with methanol and hydrogen peroxide to remove any endogenous peroxidase. The sections were treated with guanidinium hydrochloride followed by trypsin to enhance antigen detection. Then, the sections were incubated for 20 min in PBS containing 3% goat serum (Gibco, Thermo Fisher Scientific, Waltham, MA) to block non-specific binding sites. Following manufacturer instructions, the slides were incubated overnight with primary antibodies for surfactant protein B (SP-B rabbit polyclonal, 20 μg/ml, Hycult Biotech, Wayne, PA) and transforming growth factor β1 (TGF-β1, 2 ng/ml, EMD Millipore, Billerica, MA). Additional slides were incubated for 1 hr with primary antibodies for E cadherin (0.5 μg/ml, Novus Biologicals, Centennial, CO), Vimentin (1:300), Ki-67 (1:100) and β-catenin (1:50) (Dako North America Inc., Carpinteria, CA). These biomarkers were selected as indicators of epithelial cell proliferation (Ki-67) and surfactant expression by ATII cells (SP-B), to assess the reciprocal relationship between TGF-β and KGF, and to determine if the associations with EMT in mature lung (E-cadherin, vimentin, β-catenin) also applies to the EMT in pathogenesis of BPD after preterm birth., KGF expression was not evaluated because of interference from rhKGF that was administered. After washing, secondary antibodies (anti-rabbit or anti-mouse biotinylated with horseradish peroxidase) were applied for 30 min according to the primary antibody. Color was developed by 3,3'-diaminobenzidine (DAB), and the slides were counterstained with hematoxylin.

Aperio© Image Analysis Algorithm (version 9, Leica Biosystems, Wetzlar, Germany) was used to quantify IHC-stained cells. The algorithm was optimized for fetal pig lung sections stained for β-catenin, E-cadherin, Ki-67, vimentin, SP-B, and TGF-β. The algorithm classified nuclei as 0, 1+, 2+, and 3+ based on staining intensity. The percentage of positively stained nuclei, average staining intensity of positive nuclei, and percentage of nuclei in each classification were exported as Excel spreadsheets and combined into a single master file for each animal.

### Statistical Analysis

Data are presented in figures and tables as means ± SD. Categorical data were compared using non-parametric tests. Continuous variables were analyzed using ANOVA for physiologic parameters. *Post hoc* Tukey's tests were performed on continuous data. Gehan- Breslow- Wilcoxon test was used to determine survival probability, Quantitative immunohistochemistry and histology data were analyzed using a Mann-Whitney U test after testing for normality. The selected level of significance was *p* < 0.05.

## Results

**Figure 1:**
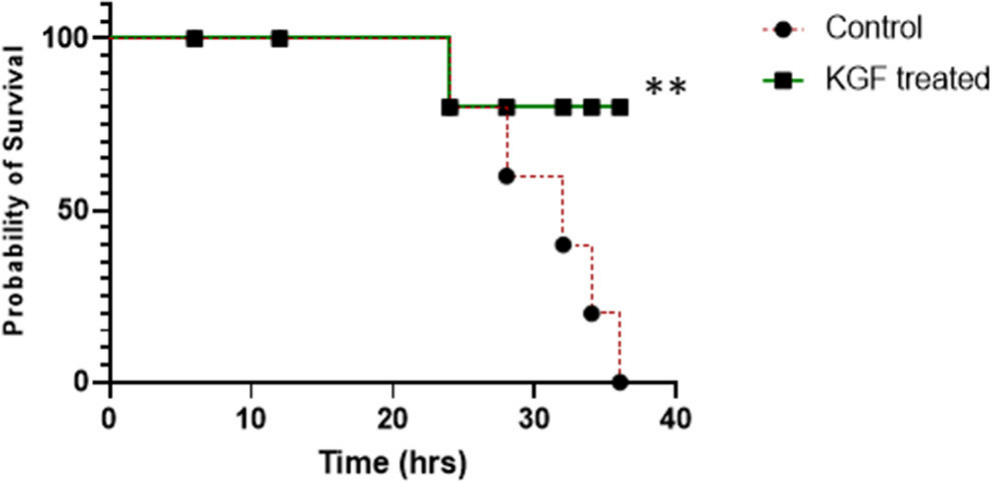
Survival plot showing survival was not different at 12 and 24 hrs between control and KGF treated groups but was significantly higher in the KGF treated group at 36 hrs (**p < 0.05).

The two treatment groups were similar in weight and proportions of males and females (Table 1). All KGF pretreated pigs and 5 of the 6 control pigs survived the 24 h of mechanical ventilation. During the 12 h of providing supplement oxygen by nasal canula, one of the KGF pigs died whereas another 4 of the control pigs died (Figure 1). The pigs that died following extubation developed worsening respiratory symptoms that consisted of chest wall retractions, poor oxygen saturation, and progressively diminishing respiratory effort, eventually leading to cardio-respiratory failure. Since the animals were not mechanically ventilated post extubation, survival to 36 h was a study outcome. The survival analysis by Gehan- Breslow- Wilcoxon test was significant with *p* ≤ 0.05 (Figure 1).

**Table 1:** Litter and group sizes, body weights, and gender distribution for preterm pigs harvested from two sows at gestational day 102.

**Litter #**	**All**	**KGF treatment**	**Control group**	***P* value**
	**(*n*, females)**	**(*n*, females)**	**(*n*; females)**	
1	945 ± 106 (12,5)	1,024 ± 43 (3, 2)	1,177 ± 49 (3,1)	NS^*^
2	972 ± 87(16, 9)	1,014 ± 38 (3, 1)	1,038 ± 20 (3, 1)	NS^*^

* *Result of Student's t-test*.

### KGF Improves Dynamic Compliance and Reduces Oxygen Requirements

**Figure 2:**
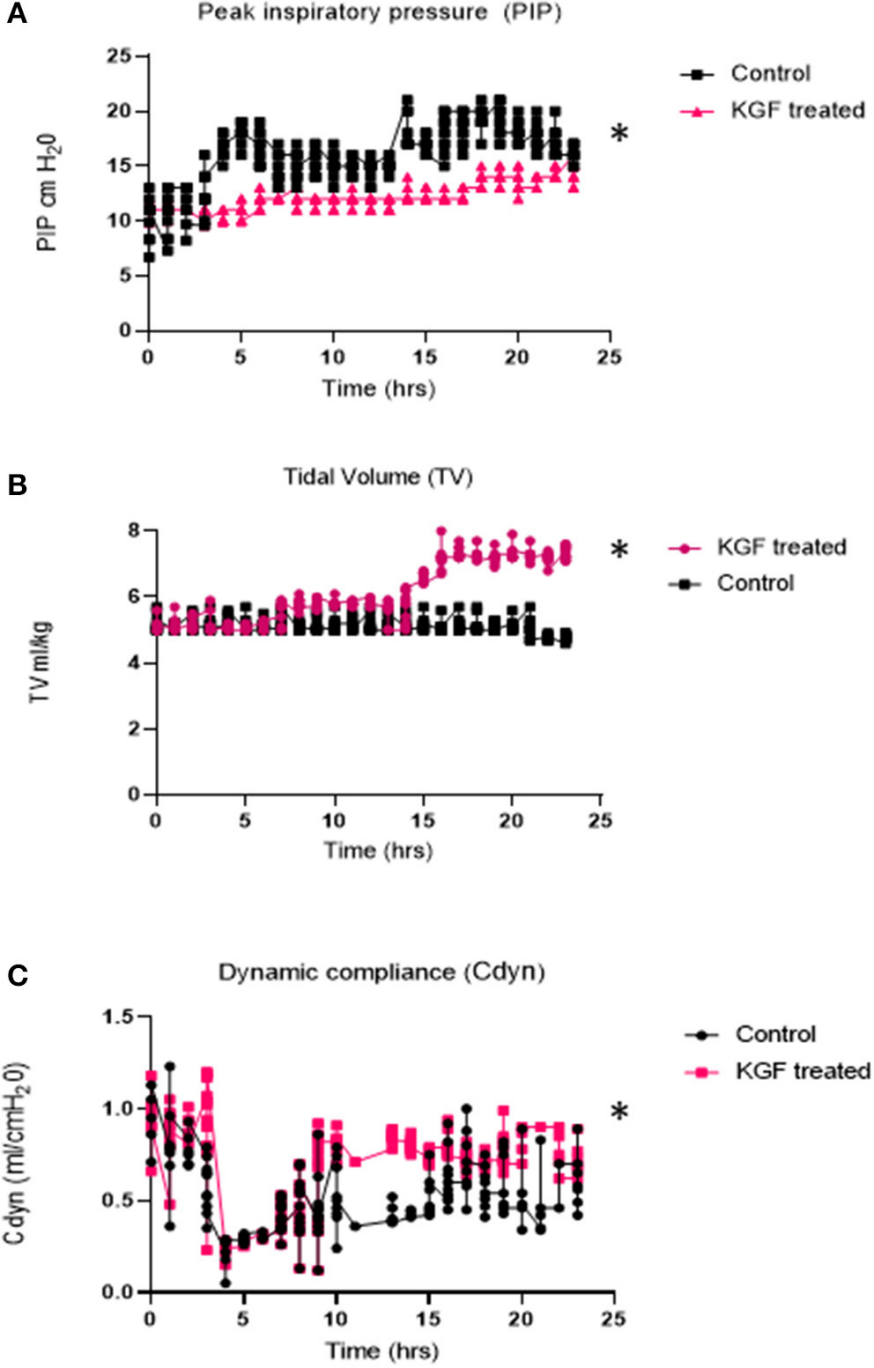
Physiologic parameters measured during 24 hr. of mechanical ventilation in KGF treated pigs (*n* = 6), control pigs (*n* = 6) **(A)** Peak Inspiratory Pressures (PIP -cm H_2_0) was significantly lower in KGF treated animals (*p < 0.05); **(B)** Tidal volumes (ml/kg) were significantly higher in the KGF treated animals (*p < 0.05); **(C)** Dynamic lung compliance (ml/cmH_2_0) was higher in KGF treated animals (*p < 0.05).

Compared with controls, KGF pretreated pigs had lower peak inspiratory pressures (PIP) (11.2 ± 1.2 vs. 16.2 ± 2.7; *p* < 0.05), (Figure 2A), higher tidal volumes (5.8 ± 2.1 vs. 5.1 ± 1.8; *p* < 0.05) (Figure 2B), corresponding with improved dynamic lung compliance (0.7 ± 0.4 vs. 0.3 ± 0.7; *p* < 0.05) (Figure 2C), and required lower FiO_2_ (58% ± 14 vs. 69% ± 12; *p* < 0.05) than control animals. Because ventilator settings were adjusted to maintain target blood gases, oxygen saturations and arterial blood gases did not differ between the control and KGF pretreatment groups during the initial 24 h of ventilation period (Table 2). These findings are consistent with the gross appearance and histopathology of the lungs at the time of death.

**Table 2:** The ventilator and blood gas data for preterm pigs provided ventilation support for 24 h.

	**KGF treatment**	**Control**	*P* value^*^
pH	7.38 (± 0.45)	7.35 (±0.55)	NS^*^
pCO_2_ (mm Hg)	42 (±24)	44 (±32)	NS^*^
paO_2_ (mm Hg)	114 (±48)	104 (±47)	NS^*^
Fraction of inspired oxygen (FiO_2_ %)	45 (±52)	55 (±45)	NS^*^
Oxygen saturation (%)	98 (±28)	97 (±37)	NS^*^
Heart rate (bpm)	164 (±57)	172 (±44)	NS^*^

*Values are Mean and (SD)*.

* *Result of Student's t-test*.

### KGF Ameliorates Lung Injury and Enhances Surfactant Expression

**Figure 3:**
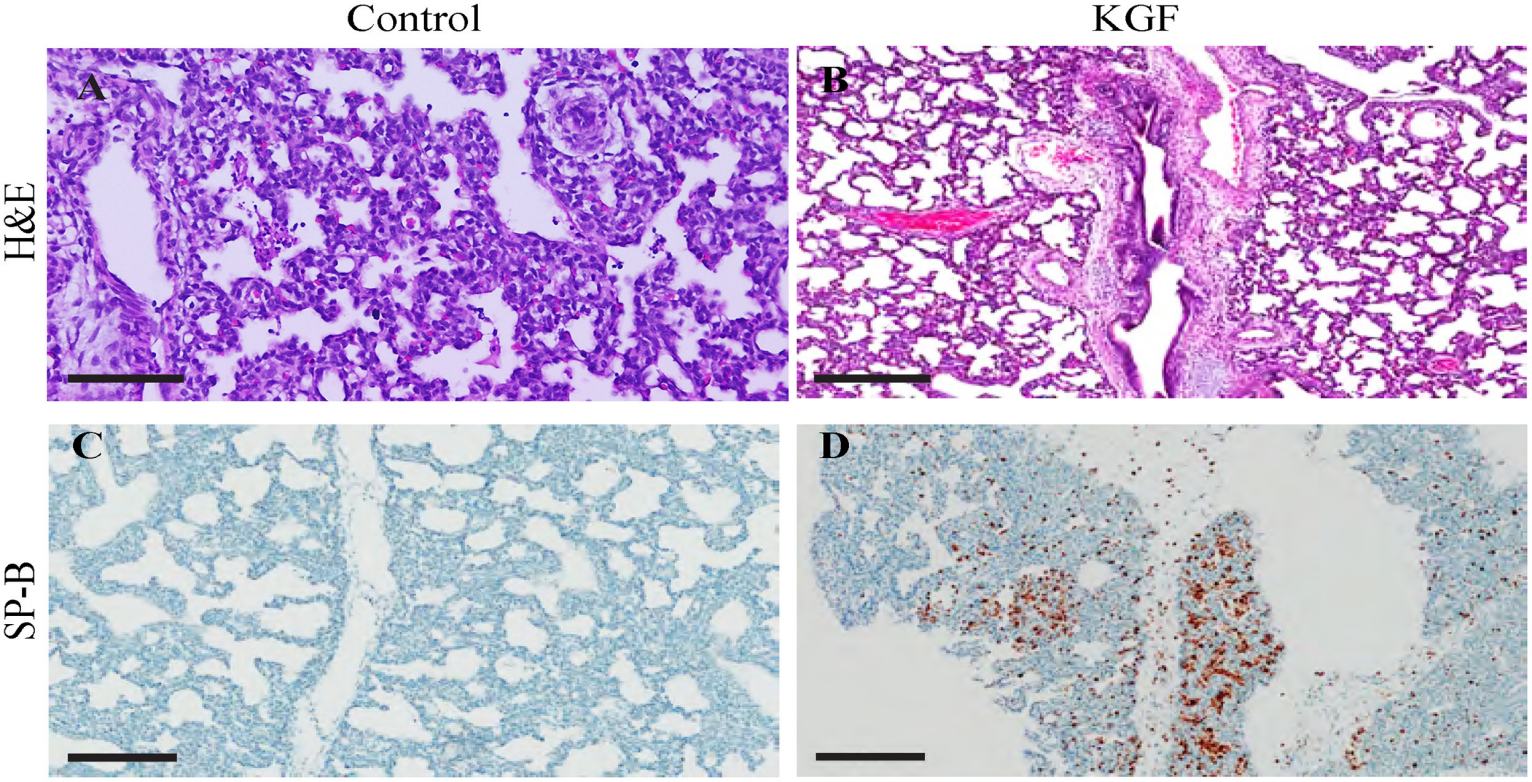
Immunohistochemistry performed on lung sections collected after 24 h of mechanical ventilation. **(A,B)** Hematoxylin and eosin (H&E)-stained sections show increased areas of inflammatory cells infiltration, edema, areas of atelectasis in control lungs compared to those in KGF lungs (Table 3, *p < 0.05) **(C,D)** Surfactant protein B (SP-B) expression is significantly higher in KGF pretreated lung tissue than in control lung tissue (19.4 ± 2.76 vs. 11.88 ± 2.30, *p* < 0.05). Scale bars in **(A,B)** are 300 μm. Other scale bars are 200 μm.

Compared with control lungs, KGF pretreated lungs showed significantly less inflammatory cell infiltrate, edema, and atelectasis (Table 3, Figures 3A,B) and significantly higher SP-B expression (19.4 ± 2.76 vs. 11.88 ± 2.30; *p* < 0.05; Figures 3C,D).

**Table 3:** Histopathology scores for the assessed parameters.

**Parameters**	**Control**	**KGF treated**
Inflammatory cell infiltrate	3.1^*^± 1.2	1.4 ± 0.7
Edema	2.1^*^± 1.6	1.7 ± 0.9
Necrosis	0	0
Atelectasis	2.2^*^± 1.4	1 ± 0.8
Hemorrhage	0	0

*Control values with an asterisk are significantly different (P < 0.05) from KGF treated pigs. Values are mean ± SD*.

* *Result of Student's t-test*.

### KGF Attenuates Epithelial Mesenchymal Transition Markers

**Figure 4:**
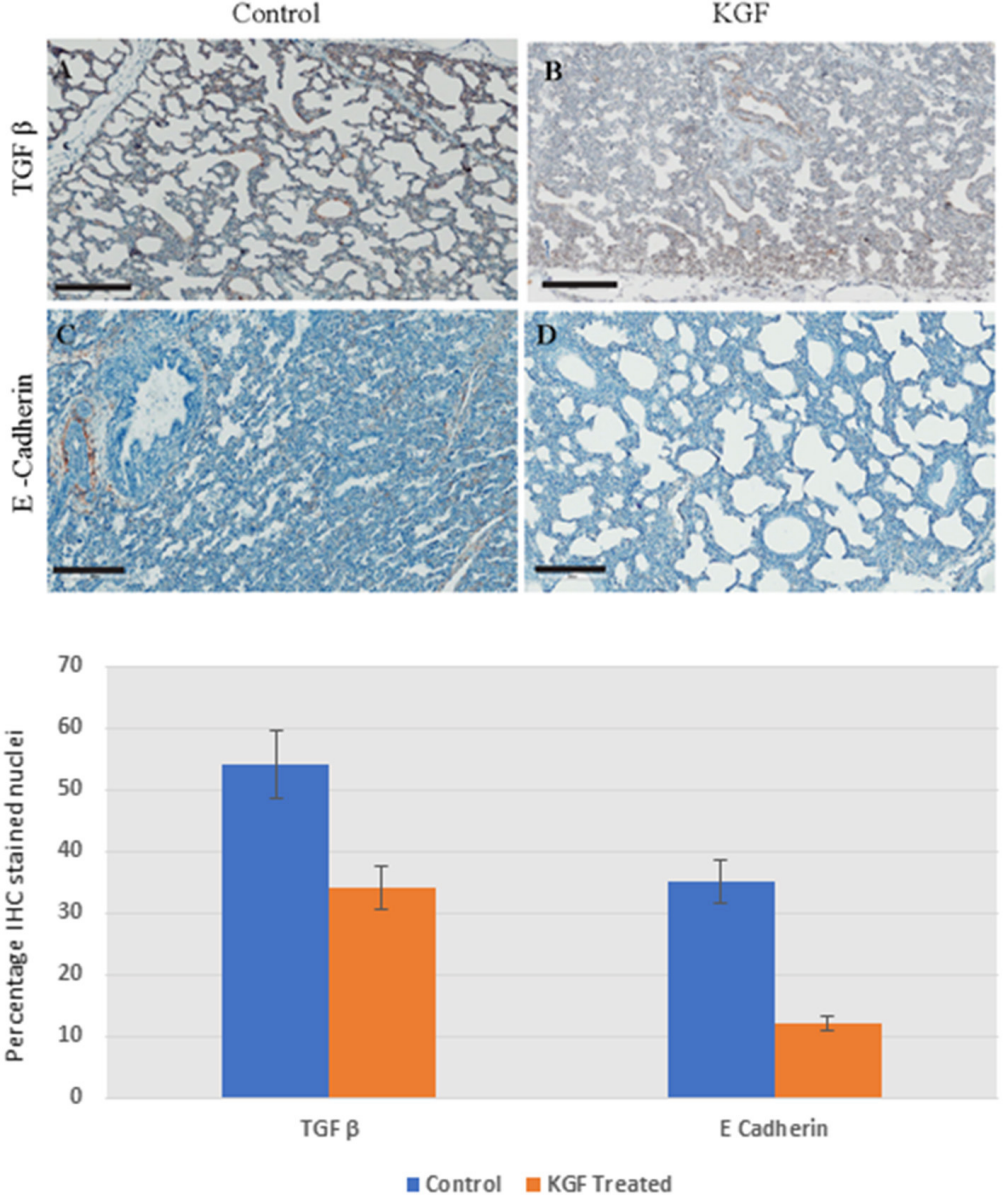
Immunohistochemistry (IHC) performed on lung sections collected after 24 h of mechanical ventilation. **(A,B)** Transforming growth factor β (TGF-β) is lower in KGF pretreated tissue than in control tissue (3.72 ± 0.08 vs. 5.31 ± 2.11, *p* < 0.05). **(C,D)** E Cadherin expression was attenuated with KGF pretreatment (1.27 ± 0.05 vs. 3.45 ± 1.81, *p* < 0.05). Scale bars are 200 μm.

**Figure 5:**
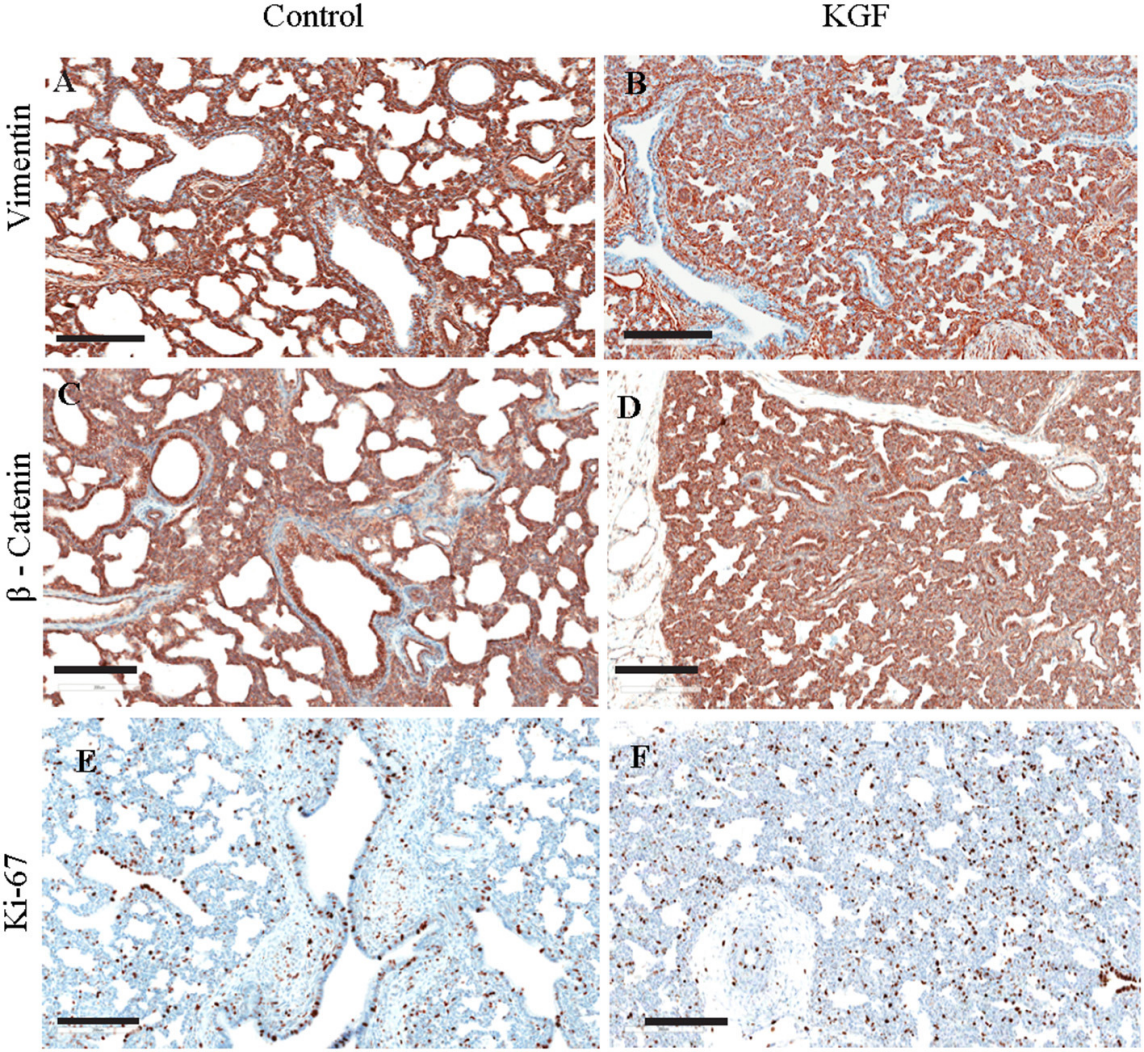
Immunohistochemistry (IHC) performed on lung sections collected after 24 h of mechanical ventilation for other EMT markers. **(A,B)** Vimentin, **(C,D)** β- Catenin, **(E,F)** Ki-67 cell proliferation IHC marker. No difference between control and pretreatment with KGF. Scale bars are 200 μm.

KGF pretreatment significantly attenuated TGF-β1 expression compared with controls (3.72 ± 0.08 vs. 5.31 ± 2.11; *p* < 0.05, Figures 4A,B). E-cadherin expression was similarly diminished in lungs of KGF treated pigs (1.27 ± 0.05 vs. 3.45 ± 1.81; *p* < 0.05). Expression of vimentin (Figures 5A,B), β-catenin (Figures 5C,D) expression and Ki-67 cell proliferation (Figures 5E,F) did not differ between the lungs of KGF and control pigs.

## Discussion

Extremely preterm pigs provide clinically relevant insights because of similarities to preterm infants that include lung anatomy and trajectory of development and compatibility with chronic *ex utero* care using neonatal intensive care protocols. Importantly, the impact of mechanical ventilation on the lungs is greater for preterm than newborn term pigs (16), causing changes in expression of proinflammatory cytokines and growth factors such as TGF-β1 and KGF (17), mimicking the greater sensitivity and responses to mechanic ventilation of preterm compared with term infants. Despite the use of clinically relevant settings and adjustments, AC+VG damages the immature lungs of preterm pigs (15), present study. The lack of exogenous surfactant administration would contribute to the diminishing compliance and acute lung injury, atelectasis, and the resulting inflammatory cell infiltrate observed in both groups. The need to increase FiO_2_ to maintain PaO_2_ within a targeted range, and particularly for the control pigs resulted in hyperoxia replicating the events that precede development of BPD among mechanically ventilated preterm infants.

Preterm infants who fail non-invasive ventilation strategies are rescued using mechanical ventilation, which reduces expression of KGF and production of surfactant protein (18). Importantly, the level of KGF expression among intubated preterm infants less than 30 weeks of gestation is inversely related with the incidence and severity of BPD (12). Although endogenous KGF was not evaluated, the intratracheal administration of rhKGF at the initiation of mechanical ventilation likely compensated for the lower expression of KGF in response to the VILI caused by the AC+VG and hyperoxia from the increase FiO2. This is consistent with the rhKGF treated pigs having greater production of surfactant, a lower extent of VILI, improved lung compliance and gas exchange allowing for lower FiO_2_, and a reduced risk of BPD. Similarly, administration of exogenous KGF to newborn rat pups exposed to hyperoxia reduces the extent of injury to the lung epithelium, enhances repair, and reduces inflammation, increases surfactant production, contributing to reduced mortality (19, 20), and stimulates surfactant production in preterm rabbit lungs (21). Although pretreatment of human volunteers with KGF before induced lung injury stimulated alveolar epithelial proliferation and production of surfactant protein D (22) the responses of preterm infants exposed to invasive mechanical ventilation are unknown. The lower morbidity and mortality of rhKGF pigs, particularly after extubation, combined with the histopathology, suggest the administration of rhKGF at the start of mechanical ventilation mitigates VILI and this may shorten the period before extubation.

Identifying injury biomarkers for the lungs of preterm infants and future risk of BPD is a research priority (7), Of the many potential candidates, TGF-β1 inhibits KGF expression (23), and reduces surfactant production in vitro by AT2 cells (24) and the higher TGF-β1 levels of the control pigs coincided with lower surfactant production. Clinically, the levels of TGF-β1 in tracheal aspirates of preterm infants are related to the extent of lung inflammation, fibrosis, abnormal lung development, and outcomes (25) and are higher among preterm infants with respiratory distress syndrome who did not receive surfactant therapy compared with infants receiving exogenous surfactant and without respiratory distress (26). It is unknown if the rhKGF administered to the pigs directly reduced TGF-β1 expression or indirectly by stimulating surfactant production, thereby reducing the severity of VILI and heightened expression of TGF-β isoforms. There is a need to define the roles of the different isoforms of TGF-α and TGF-β in mediating the responses to injurious ventilation and the responses to administering varying levels of KGF.

The prevalent models for inducing EMT and fibrosis that precede BPD (4), involve exposing neonatal term rats and near-term preterm rabbits already capable of *ex utero* life to several days of hyperoxia. The present experimental paradigm using extremely preterm pigs and intermittent positive pressure mechanical ventilation to induce VILI, leading to hyperoxia is clinically relevant to understanding the events that trigger EMT and BPD pathogenesis in preterm infants that require invasive mechanical ventilation. The higher TGF-β1 levels in the control pigs would cause a greater induction of EMT (27–29) and would be accompanied by enhanced pulmonary epithelial cell proliferation and trans differentiation into motile mesenchymal cells, and increase apoptosis of epithelial cells (22, 30). Although changes in expression of E-cadherin, vimentin, and β-catenin are known for EMT in the mature lung (31), the present findings suggest minimal or insignificant involvement with EMT during the initial 36 h after initiating injurious mechanical ventilation following extremely preterm birth. The lack of changes in collagen content in the lungs of fetal lambs after 15 days of mechanical ventilation suggest fetal lungs have a limited immediate response (32). Increased availability of species-specific immunohistochemistry markers of EMT will facilitate studies of responses of the preterm lung to rhKGF and other growth factors. Early and effective biomarkers are also needed to identify the onset of EMT, fibrosis, and initial pathogenesis of BPD.

## Conclusions

With the diminishing use of non-human primates and the limitations of neonatal rodents as translational models for preterm infants, preterm pigs represent an alternative large animal model that is clinically relevant for research of the pathogenesis of VILI and BPD and the long-term consequences. Although the small initial sample sizes and low survival of the control pigs are limitation, the approach is novel and the improved outcomes after pretreatment using rhKGF shortly after initiating mechanical ventilation suggest this represents a therapeutic strategy that may mitigate damage to the pulmonary epithelium and the resulting VILI and hyperoxia that are known risk factors for initiating BPD. The relatively short duration of the study precludes understanding if rhKGF reduces the risk of BPD. The translational extremely preterm pig model will facilitate further studies of the underlying mechanisms and signaling networks that lead to BPD pathogenesis, assist in identifying early biomarkers of lung injury, improving mechanical ventilation to reduce VILI, and for developing effective interventions for preterm infants that fail non-invasive ventilation and develop VILI.

## Data Availability Statement

The raw data supporting the conclusions of this article will be made available by the authors, without undue reservation.

## Ethics Statement

The animal study was reviewed and approved by Institutional Animal Care and Use Committees of the University of Tennessee Health Science Center (location of caesarean section) and the University of Memphis (site of ventilation and critical care) approved the protocols for the harvest, care, and sampling of preterm pigs (Sus scrofa).

## Author Contributions

RK contributed to study design, methods, data and statistical analysis, and manuscript writing. EA contributed to study methods, data collection, manuscript review and editing. CF contributed to ventilator management, animal laboratory methods and testing, ventilator data collection and analysis, and manuscript review and editing. ZJ contributed to histopathology and IHC studies, pathology data interpretation, and manuscript review and editing. RB contributed to study design, methods, animal care and sampling, data and statistical analysis, and manuscript writing. All authors contributed to the article and approved the submitted version.

## Funding

Funding was provided by a grant from Le Bonheur Children's Hospital in Memphis, TN. The Dräger VN500 mechanical ventilators used for this study were provided by Dräeger Medical, Incorporated (Dräger Medical, Incorporated, Dräger, Telford, PA).

## Conflict of Interest

CF is employed by Drager Medical Inc. The remaining authors declare that the research was conducted in the absence of any commercial or financial relationships that could be construed as a potential conflict of interest.

## Publisher's Note

All claims expressed in this article are solely those of the authors and do not necessarily represent those of their affiliated organizations, or those of the publisher, the editors and the reviewers. Any product that may be evaluated in this article, or claim that may be made by its manufacturer, is not guaranteed or endorsed by the publisher.

## Abbreviations

BPD: Bronchopulmonary dysplasia
rhKGF: Recombinant human keratinocyte growth factor
TGF-β: Tumor growth factor–β
VILI: Ventilator-induced lung injury
ATI and ATII: Alveolar type I and type II cells
EMT: Epithelial to mesenchymal transition
pNICU: Intensive care unit developed for the care of preterm pigs
UAC: Umbilical artery catheter
RDS: Respiratory distress syndrome.
